# 

**Published:** 1915-08-14

**Authors:** 


### CONTENTS.

PAGE

**Drug Values in the Treatment of Disease** ... 403

**The Training of the Health Visitor** ... ... 404

**Hospital and Institutional News ...** ... ... 405

Hospital Secretary's Death in Serbia—A Mental Hospital Convalescent Home for Soldiers—The Epping Guardians and the Local Government Board—Charing Cross Hospital and Women Students—Maternity Benefit Claims of Scottish Hospitals: Glasgow Conference—Mile End Institution for Wounded Soldiers—Canadian Convalescent Homes—The Hunt for the Early Case—The Sanatorium "Death Sentence"—The Future of Dartford Training College—The late Madame Osterberg's Work—The Supply of Salvarsan—The Empire Hospital and the War Office—The Maternity Wards at Wandsworth—Military Rank for a Lady Superintendent—A British Staff Under the Russian Red Cross—A Motor Cabman *versus* a Medical Practitioner—Mr. Punch as Hospital Critic—Phthisis and the Sanitary Inspector—The Hospital Button—Irish Guardians, Employees, and War Work—An Unwritten Chapter in Hospital History—The Institutional Side of Philanthropy—August 12—A Run of Luck to London Hospitals—This Week's Drug Market.

**Death Certificates and Insurance Companies** 411

The Companies' Attitude and the Doctor's Rights.

**County Asylums as War Hospitals** ... ... 412

By a Medical Organiser.

PAGE

**National Insurance Act Developments ...** ... 413

Arrears of Contributions and the Compulsory System.

**Medical Appointments** ... ... ... ... ... 414

**The Price of Coal (Limitation) Act, 1915** ... 415

A Hospital Superintendent's Plea for Action.

**The Religion of a Modern Doctor** ... ... 416

**Round the World** ... ... ... ... ... ... 417

Fellow-Workers and the Roll of Honour.

**King Edward's Hospital Fund for London Statistics** ... ... ... ... ... ... 418

The Valuation of Gifts in Kind.

**Frauds on German Soldiers** ... ... ... 419

**Note by a Surgeon on Active Service ...** ... 419

**Mothercraft and Child-Welfare Centres** ... 420

The Official Conditions for Obtaining Grants: By D.P.H.

**The Editor's Letter-Box** ... ... ... ... 421

Food Faddists and Something Worse.

Combined Coal and Gas Kitchener.

**Female Nurses in Male Asylum Wards** ... 421

**Bravery in the Ranks** ... ... ... ... 422

**Latest Installations in Hospital Equipment ...** 422

**The Book Market** ... ... ... ... ... 422

**The Institutional Worker** .. ... (Suppl.) 1

The Disposal of Waste Material: Answer to July Question.

Light Signals Instead of Bells: Practical Points Considered.

